# Supplementary material for: Ensemble decision of local similarity indices on the biological network for disease related gene prediction
Source: PeerJ. 2024 Sep 5;12:e17975. doi: 10.7717/peerj.17975 (PMC11380840; doi:10.7717/peerj.17975)
Supplement: Supplemental Information 1 [file peerj-12-17975-s001.docx]

**Supplementary Table 1. The all performance results of overlap analysis with GeneCard**

| **Cancer Type** | **Algorithm** | **TopN** | **Precision** | **Recall** | **F-Measure** |
| --- | --- | --- | --- | --- | --- |
| Gastric | PAC | 10 | 0,18182 | 0,02299 | 0,04082 |
|  |  | 50 | 0,08163 | 0,04598 | 0,05882 |
|  |  | 100 | 0,05714 | 0,06897 | 0,06250 |
|  | AA | 10 | 0,00000 | 0,00000 | 0,00000 |
|  |  | 50 | 0,06977 | 0,03448 | 0,04615 |
|  |  | 100 | 0,04938 | 0,04598 | 0,04762 |
|  | Jaccard | 10 | 0,00000 | 0,00000 | 0,00000 |
|  |  | 50 | 0,00000 | 0,00000 | 0,00000 |
|  |  | 100 | 0,00935 | 0,01149 | 0,01031 |
|  | RAI | 10 | 0,00000 | 0,00000 | 0,00000 |
|  |  | 50 | 0,02000 | 0,01149 | 0,01460 |
|  |  | 100 | 0,06383 | 0,06897 | 0,06630 |
| Colorectal | PAC | 10 | 0,11111 | 0,00901 | 0,01667 |
|  |  | 50 | 0,09756 | 0,03604 | 0,05263 |
|  |  | 100 | 0,06250 | 0,04505 | 0,05236 |
|  | AA | 10 | 0,10000 | 0,00901 | 0,01653 |
|  |  | 50 | 0,21739 | 0,09009 | 0,12739 |
|  |  | 100 | 0,15476 | 0,11712 | 0,13333 |
|  | Jaccard | 10 | 0,00000 | 0,00000 | 0,00000 |
|  |  | 50 | 0,01961 | 0,00901 | 0,01235 |
|  |  | 100 | 0,01786 | 0,01802 | 0,01794 |
|  | RAI | 10 | 0,40000 | 0,03604 | 0,06612 |
|  |  | 50 | 0,12245 | 0,05405 | 0,07500 |
|  |  | 100 | 0,11702 | 0,09910 | 0,10732 |
| Breast | PAC | 10 | 0,22222 | 0,01905 | 0,03509 |
|  |  | 50 | 0,11905 | 0,04762 | 0,06803 |
|  |  | 100 | 0,07229 | 0,05714 | 0,06383 |
|  | AA | 10 | 0,20000 | 0,01905 | 0,03478 |
|  |  | 50 | 0,19565 | 0,08571 | 0,11921 |
|  |  | 100 | 0,16484 | 0,14286 | 0,15306 |
|  | Jaccard | 10 | 0,00000 | 0,00000 | 0,00000 |
|  |  | 50 | 0,00000 | 0,00000 | 0,00000 |
|  |  | 100 | 0,00000 | 0,00000 | 0,00000 |
|  | RAI | 10 | 0,00000 | 0,00000 | 0,00000 |
|  |  | 50 | 0,10000 | 0,04762 | 0,06452 |
|  |  | 100 | 0,11702 | 0,10476 | 0,11055 |
| Prostate | PAC | 10 | 0,09091 | 0,00935 | 0,01695 |
|  |  | 50 | 0,03922 | 0,01869 | 0,02532 |
|  |  | 100 | 0,03175 | 0,03738 | 0,03433 |
|  | AA | 10 | 0,30000 | 0,02804 | 0,05128 |
|  |  | 50 | 0,14000 | 0,06542 | 0,08917 |
|  |  | 100 | 0,10000 | 0,09346 | 0,09662 |
|  | Jaccard | 10 | 0,00000 | 0,00000 | 0,00000 |
|  |  | 50 | 0,00000 | 0,00000 | 0,00000 |
|  |  | 100 | 0,00971 | 0,00935 | 0,00952 |
|  | RAI | 10 | 0,10000 | 0,00935 | 0,01709 |
|  |  | 50 | 0,10000 | 0,04673 | 0,06369 |
|  |  | 100 | 0,09184 | 0,08411 | 0,08780 |
| Lung | PAC | 10 | 0,30000 | 0,03093 | 0,05607 |
|  |  | 50 | 0,09804 | 0,05155 | 0,06757 |
|  |  | 100 | 0,05825 | 0,06186 | 0,06000 |
|  | AA | 10 | 0,10000 | 0,01031 | 0,01869 |
|  |  | 50 | 0,10638 | 0,05155 | 0,06944 |
|  |  | 100 | 0,09783 | 0,09278 | 0,09524 |
|  | Jaccard | 10 | 0,00000 | 0,00000 | 0,00000 |
|  |  | 50 | 0,02128 | 0,01031 | 0,01389 |
|  |  | 100 | 0,01010 | 0,01031 | 0,01020 |
|  | RAI | 10 | 0,00000 | 0,00000 | 0,00000 |
|  |  | 50 | 0,06250 | 0,03093 | 0,04138 |
|  |  | 100 | 0,06186 | 0,06186 | 0,06186 |

**Supplementary Table 2. The all performance results of overlap analysis with GO- terms**

| **Cancer Type** | **Algorithm** | **TopN** | **Precision** | **Recall** | **F-Measure** |
| --- | --- | --- | --- | --- | --- |
| Gastric | PAC | 10 | 0,597087 | 0,164879 | 0,258403 |
| Gastric | PAC | 50 | 0,531553 | 0,293566 | 0,378238 |
| Gastric | PAC | 100 | 0,449029 | 0,247989 | 0,319516 |
| Gastric | AA | 10 | 0,723577 | 0,119303 | 0,204833 |
| Gastric | AA | 50 | 0,613767 | 0,430295 | 0,50591 |
| Gastric | AA | 100 | 0,524533 | 0,601877 | 0,560549 |
| Gastric | Jaccard | 10 | 0,5 | 0,012064 | 0,02356 |
| Gastric | Jaccard | 50 | 0,594937 | 0,063003 | 0,113939 |
| Gastric | Jaccard | 100 | 0,713376 | 0,150134 | 0,248062 |
| Gastric | RAI | 10 | 0,741379 | 0,057641 | 0,106965 |
| Gastric | RAI | 50 | 0,724138 | 0,365952 | 0,486198 |
| Gastric | RAI | 100 | 0,621664 | 0,530831 | 0,572668 |
| Gastric | SMV | 10 | 0,731343 | 0,065684 | 0,120541 |
| Gastric | SMV | 50 | 0,753676 | 0,274799 | 0,40275 |
| Gastric | SMV | 100 | 0,583333 | 0,506702 | 0,542324 |
| Colorectal | PAC | 10 | 0,342105 | 0,01479 | 0,028353 |
| Colorectal | PAC | 50 | 0,637631 | 0,208191 | 0,313894 |
| Colorectal | PAC | 100 | 0,469388 | 0,183163 | 0,263502 |
| Colorectal | AA | 10 | 0,659574 | 0,070535 | 0,127441 |
| Colorectal | AA | 50 | 0,619273 | 0,445961 | 0,518519 |
| Colorectal | AA | 100 | 0,555904 | 0,605233 | 0,579521 |
| Colorectal | Jaccard | 10 | 0 | 0 | 0 |
| Colorectal | Jaccard | 50 | 0,824561 | 0,05347 | 0,100427 |
| Colorectal | Jaccard | 100 | 0,669643 | 0,085324 | 0,151362 |
| Colorectal | RAI | 10 | 0,682927 | 0,095563 | 0,167665 |
| Colorectal | RAI | 50 | 0,729358 | 0,361775 | 0,48365 |
| Colorectal | RAI | 100 | 0,668442 | 0,571104 | 0,615951 |
| Colorectal | SMV | 10 | 0,309091 | 0,01934 | 0,036403 |
| Colorectal | SMV | 50 | 0,669276 | 0,389078 | 0,492086 |
| Colorectal | SMV | 100 | 0,609598 | 0,534699 | 0,569697 |
| Breast | PAC | 10 | 0,393939 | 0,025794 | 0,048417 |
| Breast | PAC | 50 | 0,586207 | 0,151786 | 0,241135 |
| Breast | PAC | 100 | 0,537879 | 0,21131 | 0,303419 |
| Breast | AA | 10 | 0,702381 | 0,117063 | 0,20068 |
| Breast | AA | 50 | 0,564327 | 0,382937 | 0,456265 |
| Breast | AA | 100 | 0,533981 | 0,436508 | 0,480349 |
| Breast | Jaccard | 10 | 0,833333 | 0,009921 | 0,019608 |
| Breast | Jaccard | 50 | 0,666667 | 0,021825 | 0,042267 |
| Breast | Jaccard | 100 | 0,76 | 0,037698 | 0,071834 |
| Breast | RAI | 10 | 0,74359 | 0,05754 | 0,106814 |
| Breast | RAI | 50 | 0,716981 | 0,301587 | 0,424581 |
| Breast | RAI | 100 | 0,601527 | 0,390873 | 0,473842 |
| Breast | SMV | 10 | 0,590164 | 0,035714 | 0,067353 |
| Breast | SMV | 50 | 0,688841 | 0,318452 | 0,43555 |
| Breast | SMV | 100 | 0,634921 | 0,436508 | 0,517343 |
| Prostate | PAC | 10 | 0,391304 | 0,016667 | 0,031972 |
| Prostate | PAC | 50 | 0,224299 | 0,088889 | 0,127321 |
| Prostate | PAC | 100 | 0,164179 | 0,061111 | 0,089069 |
| Prostate | AA | 10 | 0,5 | 0,092593 | 0,15625 |
| Prostate | AA | 50 | 0,482456 | 0,407407 | 0,441767 |
| Prostate | AA | 100 | 0,408647 | 0,542593 | 0,466189 |
| Prostate | Jaccard | 10 | 0 | 0 | 0 |
| Prostate | Jaccard | 50 | 0,473684 | 0,033333 | 0,062284 |
| Prostate | Jaccard | 100 | 0,581081 | 0,07963 | 0,140065 |
| Prostate | RAI | 10 | 0,447761 | 0,055556 | 0,098847 |
| Prostate | RAI | 50 | 0,637427 | 0,201852 | 0,30661 |
| Prostate | RAI | 100 | 0,551351 | 0,377778 | 0,448352 |
| Prostate | SMV | 10 | 0,293103 | 0,031481 | 0,056856 |
| Prostate | SMV | 50 | 0,554795 | 0,15 | 0,236152 |
| Prostate | SMV | 100 | 0,562724 | 0,290741 | 0,383394 |
| Lung | PAC | 10 | 0,347826 | 0,024024 | 0,044944 |
| Lung | PAC | 50 | 0,523985 | 0,213213 | 0,303095 |
| Lung | PAC | 100 | 0,407018 | 0,174174 | 0,243954 |
| Lung | AA | 10 | 0,672515 | 0,172673 | 0,274791 |
| Lung | AA | 50 | 0,522852 | 0,429429 | 0,471558 |
| Lung | AA | 100 | 0,459394 | 0,569069 | 0,508384 |
| Lung | Jaccard | 10 | 0 | 0 | 0 |
| Lung | Jaccard | 50 | 1 | 0,004505 | 0,008969 |
| Lung | Jaccard | 100 | 0,74359 | 0,130631 | 0,222222 |
| Lung | RAI | 10 | 0,542857 | 0,057057 | 0,103261 |
| Lung | RAI | 50 | 0,62963 | 0,331832 | 0,434612 |
| Lung | RAI | 100 | 0,586806 | 0,507508 | 0,544283 |
| Lung | SMV | 10 | 0,537634 | 0,075075 | 0,131752 |
| Lung | SMV | 50 | 0,68125 | 0,327327 | 0,442191 |
| Lung | SMV | 100 | 0,578671 | 0,496997 | 0,534733 |
